# Investigation of maternal and perinatal outcome in a population of Iranian pregnant women infected with COVID-19

**DOI:** 10.1038/s41598-022-14112-1

**Published:** 2022-06-13

**Authors:** Soraya Saleh Gargari, Nayyereh Rahmati, Reyhaneh Fateh, Ayda Khandani, Somayeh Nikfar, Soudeh Ghafouri-Fard

**Affiliations:** 1 Men’s Health and Reproductive Health Research Center, Shahid Beheshti University of Medical Sciences, Tehran, Iran; 2 Obstetrics and Gynecology Department, Abadan University of Medical Sciences, Abadan, Iran; 3 Clinical Research Development Center of Imam Khomeini Hospital, Jiroft University of Medical Sciences, Jiroft, Iran; 4 Department of Obstetrics and Gynecology, School of Medicine, Amiralmomenin Hospital, Arak University of Medical Sciences, Arak, Iran; 5 Department of Medical Genetics, Shahid Beheshti University of Medical Sciences, Tehran, Iran

**Keywords:** Genetics, Biomarkers, Medical research

## Abstract

Infection with severe acute respiratory syndrome coronavirus 2 (SARS-CoV-2) in pregnant women might affect both maternal and neonatal outcomes. Based on the inconsistency between the results of the previous studies and the lack of data about the possible vertical transmission of SARS-CoV-2, we designed the present study to investigate the maternal and perinatal outcomes in 182 Iranian pregnant women infected with COVID-19. Among 40 PCR tests conducted on neonatal throat samples, 11 tests were positive. Among the assessed women, 22 women needed ICU admission and 30 premature labors occurred. There were significant associations between ICU admission and many parameters such as the presence of dyspnea (*P* < 0.001), COVID-19-related CT scan findings (*P* = 0.003), need for a ventilator (*P* < 0.001), and low O_2_ saturation (*P* < 0.001), all of which indicate the critical situation of patients. Notably, the cause of delivery was significantly different in both groups, with labor pain and fetal distress being the most frequent causes of delivery in non-ICU and ICU-admitted patients, respectively. Moreover, delivery route (*P* = 0.003), frequencies of IUGR (*P* = 0.042), neonatal death (*P* = 0.008) and asphyxia (*P* = 0.016), Apgar score (*P* = 0.003), and gestational age at delivery (*P* = 0.009) have been associated with ICU admission. The present investigation exhibits association between the critical situation of pregnant women affected with COVID-19 and some adverse neonatal outcomes.

## Introduction

Coronavirus disease (COVID-19) as the result of infection with the novel severe acute respiratory syndrome coronavirus 2 (SARS-CoV-2) has been rapidly disseminated throughout the world^1,2^. This disorder has also affected pregnant women and impacted pregnancy outcomes^3,4^. Pregnancy is associated with immunological alterations that might predispose women to COVID-19^5,6^. Pregnant women are vulnerable to respiratory pathogens and pneumonia because of the immunosuppressive situation and adaptive alterations existing during pregnancy. Physiological changes such as elevation of the diaphragm, elevation of oxygen consumption, and presence of edema in the respiratory tract mucosa make pregnant women intolerant to hypoxia^7^. Therefore, respiratory infections might cause a challenge to women or fetuses. Former studies have indicated the occurrence of many complications such as spontaneous abortion, premature birth, and intrauterine growth restriction in women infected with SARS^8^. The effects of COVID-19 on pregnancy outcomes have been assessed in different studies^3,4^. A retrospective study of pregnant women infected with COVID-19 in their third trimester has shown similar clinical manifestations of COVID-19 in pregnant women compared with non-pregnant affected adults. Moreover, the authors have found no evidence for the vertical transmission of viral infection in this pregnancy period^7^. Similar to those infected with SARS pneumonia, reduced fetal movement, intrauterine fetal distress, anemia, preterm labor, and multiple organ dysfunction syndrome have been reported in pregnant women infected with COVID-19^4^. Others have reported no adverse impact of COVID-19 pneumonia during pregnancy on neonatal outcomes except for a higher need for admission in the intensive care unit (ICU)^3^. Another study in a universally tested population of pregnant females with COVID-19 at delivery has reported higher rate of caesarean delivery and higher frequencies of maternal complications in the postpartum time^9^. A recent meta-analysis have shown association between maternal COVID-19 and preeclampsia, preterm birth and stillbirth^10^. Based on the inconsistency between the results of previous studies and the lack of data about the possible vertical transmission of SARS-CoV2, we designed the present study to investigate the maternal and perinatal outcomes in a population of Iranian pregnant women infected with COVID-19, in a timeframe before avaialablity of population vaccination.

## Patients and methods

### Included participants

In a cross-sectional multicenter descriptive study, pregnant women infected with COVID-19 were assessed in the period from March 2020 to July 2020. Patients were admitted in Imam Khomeini Hospital, Kerman; Ayatollah Taleghani Hospital, Abadan; Qale-e-Ganj Martyrs and Kashani Hospitals, Jiroft; 12th Farvardin Hospital, Kahnooj, and Amir-Al-Momenin Hospital, Arak. Clinical manifestations, chest CT scans, administrated therapies, and maternal and fetal outcomes were recorded in a questionnaire, retrospectively. PCR tests were conducted on symptomatic mothers. Heparin or enoxaparin was given to all patients with COVID-19 with the prophylactic dose. In ICU-admitted patients, these drugs were given with therapeutic doses. Infants were not separated from mothers. There was no limitation for breastfeeding, unless mothers were in critical conditions. Delivery route was decided based on the maternal/fetal indications. Based on the financial costs and problems with availability of PCR tests, only 40 infants were tested for COVID-19. This test was performed on the first day of life.

All methods were carried out in accordance with relevant guidelines and regulations. All experimental protocols were approved the institutional ethic committee of the Shahid Beheshti University of Medical Sciences. Informed consent has been obtained from the patints.

### Statistical analysis

Descriptive statistics were presented using frequency (percentage) and mean ± standard deviation (SD) for categorical and numerical variables, respectively. Fisher’s exact test and exact person chi-square test were used to evaluate the relationship between categorical variables. The independent t-test was used to compare the mean of numerical variables between levels of the outcome. Box plots were used to demonstrate the distribution of numerical variables in a way that facilitates comparisons across levels of a categorical variable. All analyses were performed using SPSS (version 26) and R 4.0.2. *P* values < 0.05 were considered as statistically significant.

### Ethics approval and consent to participant

All procedures performed in studies involving human participants were in accordance with the ethical standards of the institutional and/or national research committee and with the 1964 Helsinki declaration and its later amendments or comparable ethical standards. Informed consent forms were obtained from all study participants. Informed consent forms were obtained from all study participants and from legally authorised representative/next of kin of deceased patients. The study protocol was approved by the ethical committee of Shahid Beheshti University of Medical Sciences (IR.SBMU.RETECH.REC.1399.592). All methods were performed in accordance with the relevant guidelines and regulations.

## Results

### General data

A total of 182 cases were included in the study. Among 182 included cases, 158 cases were approved by the quantitative RT-PCR method, and the remaining 24 cases were PCR negative, with clinical presentations of COVID-19. Twenty-two cases were admitted to the ICU and premature birth occurred in 30 cases. The mean and SD age of women were 29.3 ± 6.15 and 30.82 ± 6.76 in non-ICU and ICU-admitted women, respectively. Gestational age at time of maternal infection was 26.83 ± 10.83 and 31.09 ± 7.93, in these two groups, respectively. Table 1 shows the baseline characteristics of the patients’ cohort.

**Table 1:** Baseline characteristics.

Variables	Levels	ICU Admission			Prematurity			Total
		No Freq (%)/mean ± SD	Yes Freq (%)/mean ± SD	*P*-value	No Freq (%)/mean ± SD	Yes Freq (%)/mean ± SD	*P*-value	Freq (%)/mean ± SD
Mother’s age		29.3 ± 6.15	30.82 ± 6.76	0.328^C^	28.83 ± 6.28	31.03 ± 6.15	0.094^C^	29.49 ± 6.23
Mother’s BMI		66.47 ± 335.13	30.7 ± 5.58	0.375^C^	26.59 ± 4.59	161.86 ± 611.38	0.323^C^	62 ± 313.43
Gestational age at infection		26.83 ± 10.83	31.09 ± 7.93	0.031^C^	29.29 ± 10.1	31.03 ± 5.38	0.205^C^	27.35 ± 10.59
Gestational age at delivery		37.6 ± 2.21	35.6 ± 3	0.009^C^	38.12 ± 1.84	34.63 ± 1.77	< 0.001^C^	37.32 ± 2.43
Birth multiplicity	Single	155 (96.88)	22 (100)	1.000^B^	110 (98.21)	27 (90)	0.063^B^	177 (97.25)
	Twin	5 (3.12)	0 (0)		2 (1.79)	3 (10)		5 (2.75)
	multiple	0 (0)	0 (0)		0 (0)	0 (0)		0 (0)

The association between categorical predictors and outcome was evaluated using A) exact Pearson chi-square test and B) fisher exact test; the mean of numeric variables was compared between outcome levels using C) independent t-test.

### Maternal characteristics and morbidity

Fever, cough, malaise, myalgia, sore throat, dyspnea, gastrointestinal (GI) symptoms, and headache were the clinical symptoms of the affected individuals. The association between ICU admission and some variables including dyspnea (*p* < 0.001), CT scan manifestations (*p* = 0.003), need for ventilator (*p* < 0.001), O_2_ saturation (*p* < 0.001), administration of antiviral drug (*p* = 0.012), administration of corticosteroid (*p* < 0.001) and administration of heparin (*p* = 0.011) were significant. Moreover, mode of delivery was significantly associated with ICU admission (*p* = 0.003) and prematurity (*p* = 0.22).

Table 2 shows maternal morbidity and complications.

**Table 2:** Descriptive statistics of maternal morbidity/complication parameters.

Variables	Levels	ICU Admission			Prematurity			Total
		No Freq (%)/mean ± SD	Yes Freq (%)/mean ± SD	*P*-value	No Freq (%)/mean ± SD	Yes Freq (%)/mean ± SD	*P*-value	Freq (%)/mean ± SD
**Maternal symptoms**								
Fever	No	48 (30)	9 (40.91)	0.331^A^	38 (33.93)	6 (20)	0.184^A^	57 (31.32)
	Yes	112 (70)	13 (59.09)		74 (66.07)	24 (80)		125 (68.68)
Cough	No	78 (48.75)	9 (40.91)	0.506^A^	54 (48.21)	13 (43.33)	0.684^A^	87 (47.8)
	Yes	82 (51.25)	13 (59.09)		58 (51.79)	17 (56.67)		95 (52.2)
Malaise	No	126 (78.75)	17 (77.27)	1.000^B^	90 (80.36)	19 (63.33)	0.056^A^	143 (78.57)
	Yes	34 (21.25)	5 (22.73)		22 (19.64)	11 (36.67)		39 (21.43)
Myalgia	No	92 (57.5)	16 (72.73)	0.247^A^	70 (62.5)	16 (53.33)	0.404^A^	108 (59.34)
	Yes	68 (42.5)	6 (27.27)		42 (37.5)	14 (46.67)		74 (40.66)
Sore throat	No	137 (85.62)	19 (86.36)	1.000^B^	98 (87.5)	24 (80)	0.374^B^	156 (85.71)
	Yes	23 (14.37)	3 (13.64)		14 (12.5)	6 (20)		26 (14.29)
Dyspnea	No	104 (65)	3 (13.64)	< 0.001^A^	68 (60.71)	14 (46.67)	0.212	107 (58.79)
	Yes	56 (35)	19 (86.36)		44 (39.29)	16 (53.33)		75 (41.21)
GI symptoms	No	149 (93.12)	21 (95.45)	1.000^B^	103 (91.96)	29 (96.67)	0.689^B^	170 (93.41)
	Yes	11 (6.88)	1 (4.55)		9 (8.04)	1 (3.33)		12 (6.59)
Headache	No	153 (96.23)	22 (100)	1.000^B^	108 (97.3)	29 (96.67)	1.000^B^	175 (96.69)
	Yes	6 (3.77)	0 (0)		3 (2.7)	1 (3.33)		6 (3.31)
**Imaging and laboratory tests**								
CT Scan	Negative	31 (29.52)	0 (0)	0.003^B^	19 (24.36)	2 (11.76)	0.345^A^	31 (24.8)
	Positive	74 (70.48)	20 (100)		59 (75.64)	15 (88.24)		94 (75.2)
PCR	Negative	21 (13.12)	3 (13.64)	1.000^B^	19 (16.96)	0 (0)	0.013^B^	24 (13.19)
	Positive	139 (86.88)	19 (86.36)		93 (83.04)	30 (100)		158 (86.81)
Echocardiography	Normal	30 (100)	8 (88.89)	0.231^B^	19 (95)	12 (100)	1.000^B^	38 (97.44)
	Abnormal	0 (0)	1 (11.11)		1 (5)	0 (0)		1 (2.56)
Ventilator	No	160 (100)	11 (50)	< 0.001^B^	107 (95.54)	25 (83.33)	0.035^B^	171 (93.96)
	Yes	0 (0)	11 (50)		5 (4.46)	5 (16.67)		11 (6.04)
O2 saturation	Less than 94%	154 (96.25)	5 (22.73)	< 0.001^B^	97 (86.61)	24 (80)	0.389^B^	159 (87.36)
	Higher than 94%	6 (3.75)	17 (77.27)		15 (13.39)	6 (20)		23 (12.64)
**Medications**								
Antiviral drug	No	97 (61.01)	7 (31.82)	0.012^A^	69 (61.61)	20 (68.97)	0.523^A^	104 (57.46)
	Yes	62 (38.99)	15 (68.18)		43 (38.39)	9 (31.03)		77 (42.54)
Antibacterial	No	15 (9.38)	1 (4.55)	0.697^B^	12 (10.71)	2 (6.67)	0.734^B^	16 (8.79)
	Yes	145 (90.62)	21 (95.45)		100 (89.29)	28 (93.33)		166 (91.21)
Corticosteroid	No	141 (89.24)	10 (47.62)	< 0.001^B^	93 (85.32)	23 (76.67)	0.274^B^	151 (84.36)
	Yes	17 (10.76)	11 (52.38)		16 (14.68)	7 (23.33)		28 (15.64)
Hydroxy-chloroquine	No	104 (65)	11 (50)	0.238^A^	74 (66.07)	24 (80)	0.184^A^	115 (63.19)
	Yes	56 (35)	11 (50)		38 (33.93)	6 (20)		67 (36.81)
Enoxaparin sodium	No	94 (58.75)	13 (59.09)	1.000^A^	68 (60.71)	27 (90)	0.004^A^	107 (58.79)
	Yes	66 (41.25)	9 (40.91)		44 (39.29)	3 (10)		75 (41.21)
Heparin	No	98 (61.25)	7 (31.82)	0.011^A^	63 (56.25)	10 (33.33)	0.039^A^	105 (57.69)
	Yes	62 (38.75)	15 (68.18)		49 (43.75)	20 (66.67)		77 (42.31)
lopinavir/ritonavir	No	110 (68.75)	11 (50)	0.094^A^	83 (74.11)	23 (76.67)	0.819^A^	121 (66.48)
	Yes	50 (31.25)	11 (50)		29 (25.89)	7 (23.33)		61 (33.52)
Plasmapheresis	No	0 (NaN)	0 (0)	–	0 (0)	0 (NaN)	–	0 (0)
	Yes	0 (NaN)	2 (100)		2 (100)	0 (NaN)		2 (100)
**Pregnancy complications**								
	No	146 (91.82)	12 (54.55)	< 0.001^B^	100 (89.29)	24 (80)	0.092^B^	158 (87.29)
	Diabetes	15 (9.43)	3 (13.64)		8 (7.14)	7 (23.33)		18 (9.94)
	Oligohydramnios	7 (4.4)	1 (4.55)		6 (5.36)	1 (3.33)		8 (4.42)
	Eclampsia	0 (0)	1 (4.55)		1 (0.89)	0 (0)		1 (0.55)
	Preeclampsia	10 (6.29)	1 (4.55)		5 (4.46)	6 (20)		11 (6.08)
	Molar	1 (0.63)	0 (0)		0 (0)	0 (0)		1 (0.55)
	ITP	1 (0.63)	0 (0)		1 (0.89)	0 (0)		1 (0.55)
	PIH	8 (5.03)	3 (13.64)		9 (8.04)	2 (6.67)		11 (6.08)
	Placental abruption	2 (1.26)	0 (0)		1 (0.89)	1 (3.33)		2 (1.1)
	HELLP	2 (1.26)	0 (0)		0 (0)	2 (6.67)		2 (1.1)
	Maternal deaths	0 (0)	7 (31.82)		3 (2.68)	2 (6.67)		7 (3.87)
	Brain stroke	0 (0)	1 (4.55)		0 (0)	0 (0)		1 (0.55)
Cause of delivery	Decreased fetal movements	5 (4.13)	0 (0)	0.029^B^	5 (4.63)	0 (0)	0.023^B^	5 (3.6)
	Spontaneous labor	45 (37.19)	3 (16.67)		38 (35.19)	10 (33.33)		48 (34.53)
	rupture of the amniotic sac	24 (19.83)	3 (16.67)		24 (22.22)	3 (10)		27 (19.42)
	Fetal Distress^&^	12 (9.92)	5 (27.78)		10 (9.26)	7 (23.33)		17 (12.23)
	Preeclampsia	12 (9.92)	1 (5.56)		7 (6.48)	6 (20)		13 (9.35)
	Oligohydramnios	8 (6.61)	1 (5.56)		6 (5.56)	2 (6.67)		9 (6.47)
	Elective abortion	12 (9.92)	2 (11.11)		14 (12.96)	0 (0)		14 (10.07)
	Cardiac arrest	0 (0)	2 (11.11)		1 (0.93)	1 (3.33)		2 (1.44)
	Uncontrolled blood sugar	1 (0.83)	0 (0)		1 (0.93)	0 (0)		1 (0.72)
	IUGR	2 (1.65)	1 (5.56)		2 (1.85)	1 (3.33)		3 (2.16)
Delivery route	NVD	63 (51.22)	3 (15)	0.003^A^	58 (51.79)	8 (26.67)	0.022^A^	66 (46.15)
	C/S	60 (48.78)	17 (85)		54 (48.21)	22 (73.33)		77 (53.85)
Spontaneous Abortion	No	128 (94.81)	21 (100)	0.595^B^	112 (100)	30 (100)	–	149 (95.51)
**Hematologic and biochemical tests**								
Leukocytes		8261.06 ± 3002.71	8940.45 ± 3624.81	0.409^C^	8690.98 ± 3210.65	8249 ± 3059.2	0.490^C^	8343.19 ± 3081.26
Lymphocyte percent		20.36 ± 8.44	18.55 ± 10.42	0.441^C^	19.95 ± 8.21	19.5 ± 9.25	0.811^C^	20.14 ± 8.69
Platelet		229,077.28 ± 79,778.09	248,909.09 ± 90,589.56	0.338^C^	239,428.57 ± 81,367.23	199,978.8 ± 76,060.67	0.016^C^	231,474.53 ± 81,148.69
AST		33.88 ± 28.83	59.7 ± 107.02	0.296^C^	39.43 ± 55.57	39.4 ± 21.56	0.996^C^	36.85 ± 45.4
Creatinine		0.76 ± 0.12	0.83 ± 0.1	0.005^C^	0.76 ± 0.13	0.81 ± 0.1	0.040^C^	0.77 ± 0.12
CRP		1.57 ± 1.17	1.63 ± 0.76	0.759^C^	1.55 ± 1.21	1.59 ± 0.94	0.844^C^	1.58 ± 1.13
ALT		28.14 ± 30.26	48.6 ± 85.12	0.299^C^	31.11 ± 44.01	31.8 ± 16.49	0.895^C^	30.49 ± 40.6
LDH		435.7 ± 179.34	594.65 ± 240.89	0.017^C^	443.85 ± 189	454.14 ± 192.6	0.800^C^	452.18 ± 192
Ferritin		60.33 ± 63.8	55 ± NA	–	78 ± 81.28	NaN ± NA	–	59.8 ± 60.17
D-dimer		1239.69 ± 1137.57	1889.55 ± 1122.82	0.125^C^	1366.67 ± 1211.04	1487 ± 1694.14	0.899^C^	1432.89 ± 1157.38
Troponins		36 ± 36.62	10 ± NA	–	28.75 ± 39.8	27.5 ± 29.15	–	34.14 ± 35.86

The association between categorical predictors and outcome was evaluated using A) exact Pearson chi-square test and B) fisher exact test; the mean of numeric variables was compared between outcome levels using C) independent t-test.

^&^ non-reassuring fetal heart tones noted on fetal heart tracing monitoring.

*GI* Gastrointestinal, *PIH* Pregnancy-induced hypertension, *IUGR* Intrauterine Growth Restriction, *FDIU* Fetal death in utero, *AST* Aspartate transaminase, *CRP* C Reactive Protein, *ALT* Alanine aminotransferase, *LDH* Lactate dehydrogenase.

### Neonatal characteristics and morbidity

There were significant associations between maternal ICU admission and IUGR (*p* = 0.042), neonatal death (*p* = 0.025), Apgar score (*p* = 0.003), asphyxia (*p* = 0.016), gestational age (*p* = 0.031), creatinine (*p* = 0.005), gestational age at delivery (*p* = 0.009) and neonatal weight (*p* = 0.036). Table 3 shows Descriptive Statistics of neonatal morbidity/complications.

**Table 3:** Descriptive statistics of neonatal morbidity/complications (These characteristics are descriptive of the entire neonatal cohort).

Variables	Levels	ICU Admission			Prematurity			Total
		No Freq (%)/mean ± SD	Yes Freq (%)/mean ± SD	*P*-value	No Freq (%)/mean ± SD	Yes Freq (%)/mean ± SD	*P*-value	Freq (%)/mean ± SD
Meconium	No	108 (87.8)	17 (89.47)	1.000^B^	96 (85.71)	29 (96.67)	0.123^B^	125 (88.03)
	Yes	15 (12.2)	2 (10.53)		16 (14.29)	1 (3.33)		17 (11.97)
IUGR	No	112 (91.06)	14 (73.68)	0.042^B^	106 (94.64)	20 (66.67)	< 0.001^B^	126 (88.73)
	Yes	11 (8.94)	5 (26.32)		6 (5.36)	10 (33.33)		16 (11.27)
FDIU	No	122 (99.19)	19 (100)	1.000^B^	111 (99.11)	30 (100)	1.000^B^	141 (99.3)
	Yes	1 (0.81)	0 (0)		1 (0.89)	0 (0)		1 (0.7)
Death within first 28 days of life	No	121 (99.18)	16 (84.21)	0.008^B^	109 (98.2)	28 (96.55)	0.504^B^	137 (97.16)
	Yes	1 (0.82)	3 (15.79)		2 (1.8)	1 (3.45)		4 (2.84)
Admission to NICU	No	98 (80.99)	9 (52.94)	0.025^B^	96 (88.07)	11 (37.93)	< 0.001^A^	107 (77.54)
	Yes	23 (19.01)	8 (47.06)		13 (11.93)	18 (62.07)		31 (22.46)
Apgar score (5 min)	≥ 7	120 (98.36)	15 (78.95)	0.003^B^	108 (97.3)	27 (90)	0.128^A^	135 (95.74)
	< 7	2 (1.64)	4 (21.05)		3 (2.7)	3 (10)		6 (4.36)
Asphyxia	No	121 (100)	16 (88.89)	0.016^B^	109 (98.2)	28 (100)	1.000^B^	137 (98.56)
	Yes	0 (0)	2 (11.11)		2 (1.8)	0 (0)		2 (1.44)
UCABG	NL	11 (100)	5 (100)	–	12 (100)	4 (100)	–	16 (100)
Sepsis	No	122 (100)	15 (100)	–	109 (100)	28 (100)	–	137 (100)
	Yes	0 (0)	0 (0)		0 (0)	0 (0)		0 (0)
Respiratory distress	No	117 (96.69)	14 (93.33)	0.448^B^	104 (96.3)	27 (96.43)	1.000^B^	131 (96.32)
	Yes	4 (3.31)	1 (6.67)		4 (3.7)	1 (3.57)		5 (3.68)
Nasopharyngeal COVID-19 PCR test	Negative	25 (78.12)	4 (50)	0.182^B^	19 (70.37)	10 (76.92)	1.000^B^	29 (72.5)
	PCR positive	7 (21.88)	4 (50)		8 (29.63)	3 (23.08)		11 (27.5)

The association between categorical predictors and outcome was evaluated using (A) exact Pearson chi-square test and (B) fisher exact test; the mean of numeric variables was compared between outcome levels using (C) independent t-test.

*IUGR* Intrauterine Growth Restriction, *FDIU* Fetal death in utero, *UCABG* Umbilical cord arterial blood gas.

Eleven out of 40 infants tested for COVID-19 had positive PCR results. None of them had symptoms related with COVID-19 at the first day of life. Significant associations were also observed between prematurity and variables including positive maternal PCR results (*p* = 0.013), need for ventilator (*p* = 0.013), lack of administration of enoxaparin sodium (*p* = 0.004), administration of heparin (*p* = 0.039), diabetes (*p* = 0.018), preeclampsia (*p* = 0.012), cause of delivery (*p* = 0.023), delivery route (*p* = 0.022), IUGR (*p* < 0.001), admission to NICU (*p* < 0.001), platelet count (*p* = 0.016), creatinine level (*p* = 0.040), gestational age at delivery (*p* < 0.001), and neonatal weight (*p* < 0.001).

**Figure 1:**
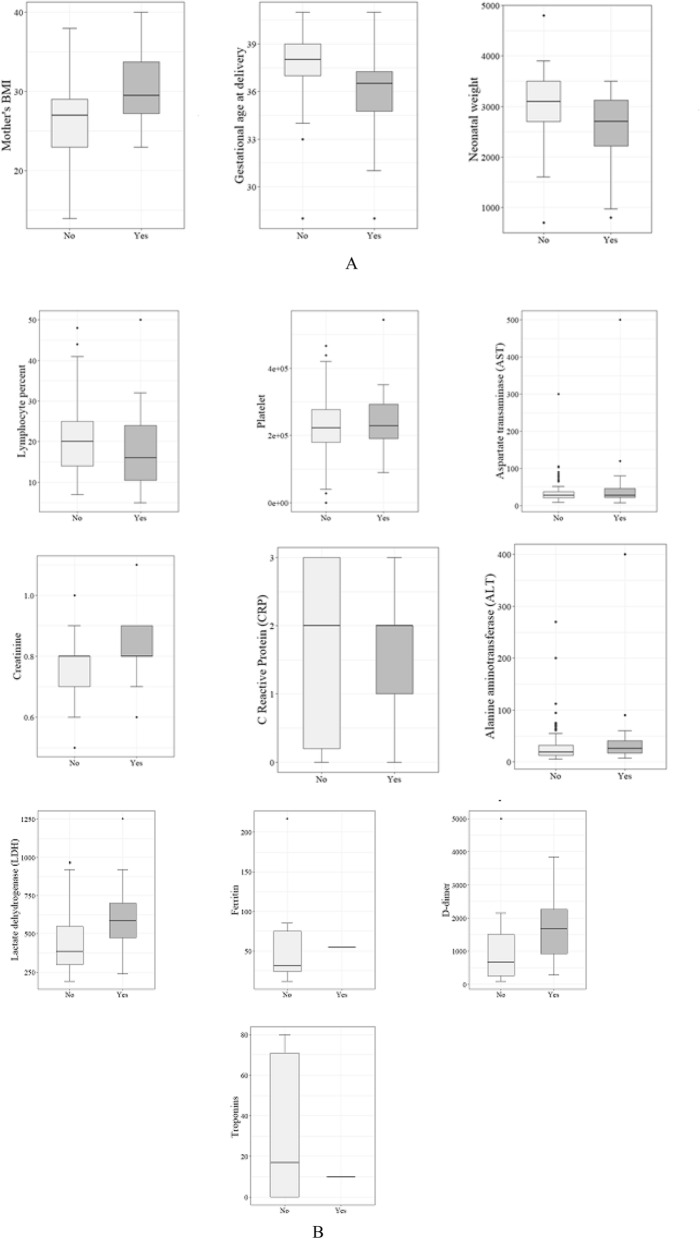
The distribution of variables among ICU admission levels [(**A**): maternal/neonatal characteristics, (**B**): Laboratory tests].

**Figure 2:**
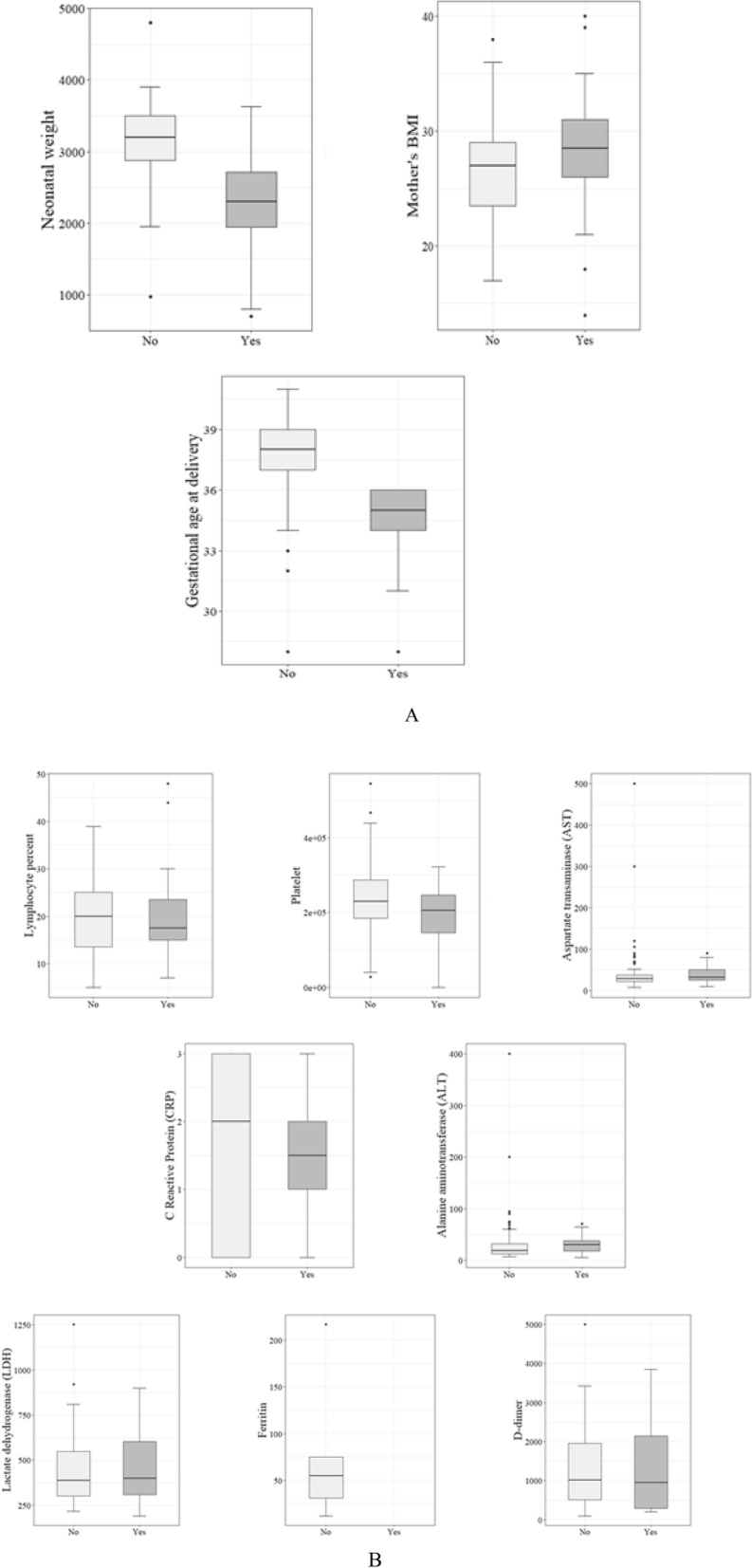
The distribution of variables among prematurity levels [(**A**): maternal/neonatal characteristics, (**B**): Laboratory tests].

In the next step, boxplots were depicted to indicate how the values in the numeric variables are spread out in ICU admission and at premature levels (Figs. 1 and 2).

## Discussion

Pregnancy is regarded as a distinctive immunological situation. During pregnancy, the maternal immune system is expected to establish and maintain tolerance to the fetus which is regarded as an allogenic graft, whereas it should preserve the aptitude for protection against pathogens. Therefore, both systemic and local immune responses should be finely regulated during pregnancy^5^. Changes in the immune responses during pregnancy might make pregnant women susceptible to COVID-19^5^ and lead to perinatal and maternal complications. In the present study, we investigated these complications in a large cohort of pregnant women affected with COVID-19. Globally, the clinical manifestations of COVID-19 were not different between our cohort of pregnant women and previously reported cases^4^. Among the assessed women, 22 women needed ICU admission and 30 premature labors occurred. However, there was no significant difference in the rate of prematurity between ICU-admitted and the other group of pregnant women. There were significant associations between ICU admission and many parameters such as the presence of dyspnea, COVID-19-related CT scan findings, need for a ventilator, and low O_2_ saturation—all of which indicate the critical situation of patients. Consistent with the difference in the therapeutic protocols for patients admitted in the ICU and those being treated in general wards, ICU admission was associated with administration of antiviral drugs, corticosteroids, and heparin treatments.

Notably, the cause of delivery was significantly different between the two groups with preterm labor pain and fetal distress being the most frequent cause of delivery in non-ICU and ICU-admitted patients, respectively. This might indicate the impact of a critical situation of the pregnant women on fetal distress. Moreover, we reported that delivery route, neonatal death and asphyxia, Apgar score, and gestational age at delivery have been associated with ICU admission. However, the rate of IUGR was lower in the non-ICU population. This might be due to better maternal health conditions in non-ICU admitted women compared to ICU-admitted ones.

The observed association between ICU admission and the delivery route is consistent with the formerly reported need for emergency C-sections as a pregnancy complication in women affected with COVID-19^11^, MERS^12^, and SARS infections^13^. Based on a recent meta-analysis, symptomatic COVID-19 has been associated with higher possibility of C-section and preterm birth when compared to asymptomatic infection^10^.

The frequency of preterm labor in our cohort of patients was significantly lower than the previously reported rate of 42%^14^. A population-based cohort study has suggested an association between COVID-19 during the late pregnancy and higher risk of iatrogenic preterm birth^15^.

In the current study, significant associations have also been observed between prematurity and variables including positive PCR results, need for a ventilator, lack of administration of enoxaparin sodium, administration of heparin, diabetes, preeclampsia, delivery route, platelet count and creatinine level. These observations indicate possible link between prematurity and maternal health complications. Moreover, many parameters such as diabetes and preeclampsia might affect the perinatal complications in women affected with COVID-19.

Regarding adverse neonatal outcomes, ICU admission was associated with a low Apgar score and admission of the neonate in the neonatal ICU (NICU) ward. A systematic review of clinical outcomes of 211 PCR‐confirmed and 84 clinically diagnosed cases of pregnant women affected with COVID‐19 has reported the admission of almost one‐third of neonates in the NICU^16^. Yet, in our cohort of patients, 31 cases were admitted to the NICU.

In our cohort of patients, asphyxia occurred in two cases; both of them were born to ICU-admitted pregnant women. This observation might also imply the impact of a critical situation of the mother on the neonate. Among 40 PCR tests conducted on neonatal throat samples, 11 tests were positive, indicating the possible transmission of SARS-CoV-2. Consistent with our finding, Zeng et al. have reported three cases of positive SARS-CoV-2 among 33 neonates born to women affected by COVID-19^17^. Although contamination from the environment cannot be ruled out, similar to the study conducted in China^17^, maternal origin is mostly supported because of strict prevention measures. A systematic review of literature has indicated a 3.2% rate of vertical transmission of SARS-CoV-2^18^.

As expected, significant associations have also been observed between prematurity and IUGR, admission to NICU, and neonatal weight.

The data presented above shows that neonatal outcomes are different in this cohort of pregnant women infected with COVID-19. The observed differences in the neonatal outcomes might be explained by the complexity of immune responses, differences in gestational age, and the duration and severity of COVID-19 infection, necessitating personalized approaches for the treatment of these women.

Taken together, in the present study, we have reported the association between many clinical variables and perinatal outcomes in pregnant women affected with COVID-19. The main strength of the present study is the inclusion of a large sample size of cases which is comparable with the published systematic reviews in this field.

## Data Availability

The analysed data sets generated during the study are available from the corresponding author on reasonable request.

## Abbreviations

SARS-CoV-2: Severe acute respiratory syndrome coronavirus 2
COVID-19: Coronavirus disease
ICU: Intensive care unit
SD: Standard deviation
GI: Gastrointestinal
NICU: Neonate in the neonatal ICU
